# Study on the evolutionary game of the three parties in the combined medical and health-care PPP project

**DOI:** 10.3389/fpubh.2023.1072354

**Published:** 2023-01-26

**Authors:** Min Zhang, Ping Yao

**Affiliations:** College of Economics and Management, Nanjing Forestry University, Nanjing, China

**Keywords:** combined medical and health-care PPP, evolutionary game, government regulation, Matlab simulation, integration of medical and health-care

## Abstract

In the context of ageing, the combination of medical and health-care has become a major trend, and the introduction of the PPP model into combined medical and healthcare projects can solve the problem of lack of funds for the development of combined medical and health-care projects. Based on the evolutionary game theory, this paper constructs a three-party evolutionary game model to study the strategy choice of each party in the game process, and also analyses the evolutionary equilibrium strategy and the impact of parameter adjustment on the evolutionary strategy under different situations. The results show that the optimal solution is for the private sector to choose to provide high quality services, for the government to choose to actively regulate and for the public to choose to actively monitor. For each player, the incentives and disincentives influence their behavioural strategies. Based on the above analysis, this paper suggests establishing an independent regulator, broadening public feedback channels, and improving PPP-related laws, as well as innovating project operation methods and improving enterprise operational capabilities.

## 1. Introduction

At this stage, the combination of medical services and elderly care services has become a new mode of supply in the elderly care market. The Opinions on Strengthening Aging Work in the New Era promulgated by the Central Committee of the Communist Party of China and the State Council in November 2021 clearly states, “We should deeply promote the integration of medical care and elderly care, encourage medical institutions to cooperate with elderly care institutions, further integrate and optimize grassroots medical and elderly care resources, and provide medical assistance and rehabilitation and care services for the elderly” (1). The development of combined medical and health-care service system will be a long-term, systematic and comprehensive public health construction work, with huge capital requirements. Relying solely on the government's financial input cannot fully meet the development capital needs, and the reasonable use of social capital participation is an effective way to solve the problem.

The application of PPP projects in the field of medical and health-care can not only effectively relieve the financial pressure of the government, but also inject fresh blood into the development of combined medical and health-care projects. Regarding the application of health-care PPP in China, as of 19 September 2022, the number of PPP projects in the PPP project pool has reached 10,272, with a total amount of 1,644.7 billion yuan, which to some extent highlights the widespread application of the PPP model in major fields. Among them, there are now a total of 43 combined medical and health-care PPP projects, accounting for only 0.42% of the total number of PPP projects in the pool (2), which indicates that the implementation of combined medical and health-care PPP projects in China is still in its initial stage. Therefore, domestic experts and scholars mainly focus on the necessity and feasibility of introducing the PPP model in the field of combined health-care (3–14), as well as the countermeasures to the problems of combined medical and health-care PPP projects.

Regarding the necessity of introducing the PPP model in the field of medical and health-care combination, it provides new ideas for the development of medical and health-care combination, as complete public ownership of medical and health-care services would greatly increase the financial burden of government departments, while complete privatization would lead to market failure due to the nature of public goods in medical and health-care services (3). In view of the lack of healthcare resources and elderly resources in remote areas of developing countries, the combined medical and health-care PPP model can provide relatively stable financial support and professional talents, which can effectively solve the difficult problems faced by developing countries (4, 5). In order to study the feasibility of introducing PPP model in the field of medical and health-care combination, some scholars introduced TOT (Transfer-Operate-Transfer) model into combined medical and health-care projects and found that the financing efficiency and construction quality of the projects were significantly improved, thus arguing the feasibility of introducing PPP model in the field of medical and health-care combination (6). In addition, some scholars found that the total benefits of the project were greater than the total costs by measuring in the specific combined medical and health-care PPP projects, thus again arguing the feasibility of introducing PPP projects in the combined medical and health-care field (7).

Regarding the research on the problems and countermeasures in the development of combined medical and health-care PPP projects, some scholars have found the following problems, such as high barriers to social capital participation, inefficient management, and limited channels for operating returns (8, 9). Unequal distribution of resources, single funding channel and lack of talents have become key factors limiting the further development of combined medical and health-care PPP projects (10). In response to the above problems, scholars have provided answers from the government's perspective and social capital's perspective, respectively. From the government's perspective, some scholars believe that laws and regulations in the field of combined medical and health-care PPP should be further established, and the risk-sharing and return mechanisms should be further changed to improve the investment and financing environment (11, 12). From the perspective of social capital, some scholars believe that exploring new profitability channels is important for the development of combined medical and health-care PPP, while proposing the establishment of an effective internal management system so as to attract more talents (13, 14).

The above problems are all focused on the theoretical level, at practical level, the government, as the leader and participant in combined medical and health-care PPP projects, and also the “person in charge” in the minds of the public, is not involved in the specific operation of combined medical and health-care PPP projects, but is more of a regulator. The absence of government in practice may result in existing government monitoring systems that do not effectively capture the value of projects.

To address the issues that arise at the practice level, good governance and regulatory quality become necessary conditions required for the development of combined medical and health-care PPP projects (15). With more and more experts and scholars conducting in-depth research on PPP model-related issues, the method of evolutionary game is gradually applied to PPP model (16). At present, evolutionary games are mostly applied to transportation and environmental protection PPP projects, and the evolutionary game approach is used to discuss the cooperation mechanism and supervision quality of the projects (16). Unlike transportation and environmental protection PPP projects, where hard indicators can be used to measure the quality of supervision, combined medical and health-care PPP projects mainly provide soft services, so how to regulate combined medical and health-care PPP projects has become a major problem for government departments (16–19). Based on the evolutionary game theory, this paper constructs a three-party evolutionary game model, studies the strategy choice of each party in the game process, analyzes the evolutionary equilibrium strategy under different situations and the influence of the adjustment of parameter on the evolutionary strategy, and puts forward corresponding suggestions and countermeasures from the level of government regulation, in order to design the regulatory mechanism of combined medical and health-care PPP projects and attract social capital to join the healthcare industry. The purpose of this paper is to provide theoretical reference for the government to design the regulatory mechanism of combined medical and health-care PPP projects and to attract social capital to join the elderly industry by choosing the PPP model.

## 2. Hypothesis

It is hypothesized that the three parties in the game, namely the government, the private sector and the public, are all finite rational persons who do not fully understand each other's strategies and efficiency functions, and that the strategies of the three parties in the game are constantly being adjusted until the optimal strategy is found. It is hypothesized that the government department aims to achieve the social interest and the private sector and the public aim to maximize their own interests (20).

The related gains and losses in the private sector are as follows:

It is hypothesized that the probability of the private sector providing a high quality service (no default) is *x* and the probability of providing a low quality service (default) is 1 − *x*.
If the private sector chooses to provide high quality services, the minimum benefit based on user fees is *Mi*, the cost to be paid is *CH*, and the operating subsidy available after government assessment is *W*_*e*_. The level of reputation given by the public when the private sector provides high quality services is α1.
If the private sector provides low quality services, the minimum benefit under user fees is *Mi*, the cost to the private sector is *CL*, and in conjunction with the above we know that *CH* > *CL*. However, if the private sector is found to be monitored by the government or reported by the public, the private sector is penalized and the resulting loss is *Ws*. The level of reputation given by the public when the private sector provides low quality services is α2, and in conjunction with the above we know that α1 > α2.

The related gains and losses in the government sector are as follows:

It is hypothesized that the probability of a government department choosing to regulate is *y*, and the probability of choosing not to regulate is 1 − *y*.
If the government chooses to regulate, the fixed benefit is *Fi*, the cost to the government is *Cg*, and the additional benefit is *Rg* (the additional rewards from a higher authority and public recognition for monitoring private sector default), the regulatory to private sector default collection fines is *Ws*. The level of reputation given by the public when the government chooses to regulate is β1.
If the government chooses not to regulate, it will not incur regulatory costs and will not receive additional rewards from a higher authority or fines collected. At this point, if the government is censured by a higher authority or complained by the public for failing to regulate in a timely manner and thus not detecting a private sector default, the loss incurred is *Fg*. The level of reputation given by the public in the absence of government oversight is β2, which, in conjunction with the above, we know that β1 > β2.

The relevant gains and losses to the public are as follows:

It is hypothesized that the probability of public participation in monitoring the combined medical and health-care PPP project is *z*, and the probability of not monitoring is 1 − *z*.
If the public chooses to monitor, the cost to the public is *Cp*, and the rewards from the government is *Rp*. When the private sector provides low quality health care services, the public suffers a loss of *R*1, and the compensation for reporting the damage is *P*.
If the public chooses not to monitor, they incur no monitoring costs and receive no rewards from government departments. However, if the private sector provides a low quality service at this point, the public suffers a loss of *R*1.
It is hypothesized that all public complaints against the private sector are genuine and valid, and that the government will always be able to detect irregularities (provide low-quality services) in the private sector before the public, provided it takes its regulatory role seriously.
It is hypothesized that if the government chooses to regulate, it will inevitably detect defaults in the private sector, or not if it chooses not to.

The above specific parameters and their meanings are shown in the Table 1.

**Table 1:** Parameters and meaning of game behavior.

**Parameters**	**Meaning**
*M*_*i*_	Minimum benefits to the private sector based on user fees
*CH*	The cost to the private sector of choosing to provide quality services
*We*	Operating subsidies available to the private sector following government assessment of quality service delivery
α1	Level of reputation given by the private sector when providing high quality services under public scrutiny
*CL*	The cost to the private sector of choosing to provide low-quality services
*Ws*	Losses arising from government oversight or public reporting of low-quality services provided by the private sector
α2	Level of reputation given by the private sector when providing low quality services under public scrutiny
*Fi*	Government sector fixed income
*Rg*	Additional benefits gained by government departments choosing to regulate
*Cg*	Cost of active government regulation
β1	Government departments actively regulate the level of reputation given to the government when under public scrutiny
*Rp*	Rewards given by government departments to people who actively monitor
*Fg*	Penalties for government failure to regulate in a timely manner so that violations by the private sector that were not detected by the public are discovered
β2	Government departments do not actively regulate the level of reputation given to the government by public scrutiny
*Cp*	Costs incurred when the public actively participates in monitoring
*R*1	Damage to the public when the private sector provides low-quality services
*P*	Compensation for the public in case of damage due to reporting

Based on the above hypothesis, the evolutionary game payment matrix between the government apartment the private sector and the public is shown in the Table 2.

**Table 2:** Government, private sector, and public payment-revenue matrix 3 results.

**Game participants**				**Government regulators**	
				**supervision** *y*	**No supervision** 1 − *y*
Private sector	No violation *x*	Public	Participation *z*	*Mi* + *We* + α1 − *CH*, *Fi* + *Rg* + β1 − *Cg* − *Rp*, *Rp* − *Cp*	*Mi* + *We* + α1 − *CH*, *Fi* + β2 − *Rp*, *Rp* − *Cp*
			No participation 1 − *z*	*Mi* + *We* − *CH*, *Fi* + *Rg* − *Cg*, 0	*Mi* + *We* − *CH*, *Fi*, 0
	violation 1 − *x*	Public	Participation *z*	*Mi* + α2 − *CL* − *Ws*, *Fi* + *Rg* + *Ws* + β1 − *Cg* − *Rp* *Rp* − *Cp* − *R*1 + *P*	*Mi* + α2 − *CL*, *Fi* + β2 − *Rp* − *Fg*, *Rp* − *Cp* − *R*1 + *P*
			No participation 1 − *z*	*Mi* − *CL* − *Ws*, *Fi* + *Rg* + *Ws* − *Cg*, −*R*1	*Mi* − *CL*, *Fi*, −*R*1

## 3. Model analysis

### 3.1. Theoretical analysis

Game theory is the study of the decision-making behavior of intelligent decision-makers in situations of interest. Game theory, also known as response theory, is a branch of modern mathematics and an important part of operations research (21). In practical terms, there are a number of conflicting or related subjects, known as decision makers or game players, who are able to make decisions on their own. Game theory is the abstraction of real problems and the creation of corresponding mathematical models to analyze, predict and even intervene in the decisions or behavior of individuals. These models may be out of touch with reality, but they make it easier to interpret actual competitive situations or social dilemmas such as conflict and cooperation than complex real-life situations (21).

In 1950, the American mathematician Nash (22) introduced an important concept in game theory, the Nash Equilibrium. That is, when the individuals involved in the game are in Nash equilibrium, no individual can gain more by unilaterally changing his or her strategy. In practice, however, the assumptions of perfect rationality and perfect information of the participating individuals are difficult to satisfy. In 1973, ecologists Smith and Price published their famous paper “The Logic of Animal Conflict” (23), which combined biological evolution and classical game theory and proposed the Evolutionary Stable Strategy (ESS) based on the study of eco-evolutionary phenomena. Evolutionary Stable Strategy (ESS) is an important concept in evolutionary game theory, which also marks the birth of evolutionary game theory (23).

Evolutionary game theory embodies the idea of biological evolution, the so-called 'survival of the fittest', and the Nash equilibrium of classical game theory is reflected in evolutionary stable strategies (ESS) in evolutionary games. A strategy is evolutionarily stable if all individuals in the group adopt a certain strategy and no mutation strategy adopted by a small group of other individuals can invade the group (21).

### 3.2. Analysis of the stability of the private sector strategy

The expected benefits of providing high quality services or providing low quality services in the private sector, as well as the average expected benefits are:


$$\begin{array}{l} Y11=yz\left(Mi+We+\alpha 1-CH\right)+\left(1-y\right)z\left(Mi+We+\alpha 1-CH\right)\\ \;+y\left(1-z\right)\left(Mi+We-CH\right)\\ \;+\left(1-y\right)\left(1-z\right)\left(Mi+We-CH\right)\\ Y12=yz\left(Mi+\alpha 2-CL-Ws\right)+\left(1-y\right)z\left(Mi+\alpha 2-CL\right)\\ \;+y\left(1-z\right)\left(Mi-CL-Ws\right)+\left(1-y\right)\left(1-z\right)\left(Mi-CL\right)\\ \bar{Y1}=xY11+\left(1-x\right)Y12 \end{array}$$

The replication dynamics equation for the private sector is:


$$\begin{array}{l} Y\left(x\right)=dx/dt=x\left(Y11-\bar{Y1}\right)=x\left(x-1\right)\\ \;\left[CH-CL-We-z\left(\alpha 1-\alpha 2\right)-yWs\right] \end{array}$$

The first-order derivatives of *x* and the set *G*(*y*) are:


$$\begin{array}{l} d\left(Y\left(x\right)\right)/dx=\left(2x-1\right)\left[CH-CL-We-z\left(\alpha 1-\alpha 2\right)-yWs\right]\\ \;G\left(y\right)=CH-CL-We-z\left(\alpha 1-\alpha 2\right)-yWs \end{array}$$

**Figure 1:**
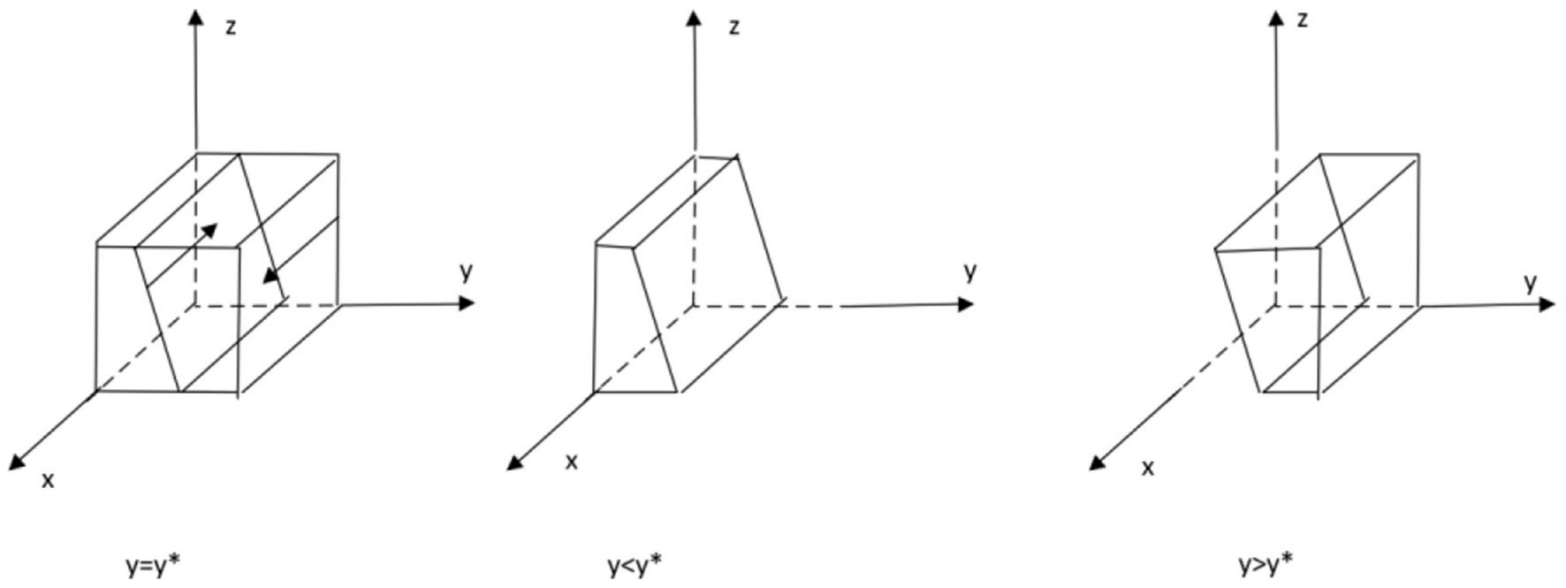
Phase diagram of the evolution of private sector strategies.

From the stability theorem of the differential equation, it follows that the probability of the private sector choosing to provide high quality services is in a steady state must satisfy:*Y*(*x*) = 0 and *d*(*Y*(*x*))/*dx* < 0. Since ∂*G*(*y*)/∂*y* < 0, *G*(*y*) is a decreasing function with respect to *y*. Thus, when *y* = [*CH* − *CL* − *We* − *z*(α1 − α2)]/*Ws* = *y*∗, *G*(*y*) = 0, at which point *d*(*F*(*x*))/*dx* ≡ 0, the private sector cannot determine a stabilization strategy. When *y* < *y*∗, *G*(*y*) > 0, at this time *d*(*Y*(*x*))/*dx*|*x* = 0 < 0, at this time, *x* = 0 is the evolutionary stabilization strategy for the private sector. Conversely when *y* > *y*∗, *G*(*y*) < 0, *d*(*Y*(*x*))/*dx*|*x* = 1 < 0, at this point, *x* = 1 is the evolutionary stable strategy for the private sector. The evolutionary phase diagram of the private sector's strategy is shown in Figure 1.

### 3.3. Analysis of the stability of government sector strategies

The expected benefits of government sector regulation, non-regulation and the average expected benefits are:


$$\begin{array}{l} Y21=xz\left(Fi+Rg+\beta 1-Cg-Rp\right)+x\left(1-z\right)\left(Fi+Rg-Cg\right)\\ \;+\left(1-x\right)z\left(Fi+Rg+Ws+\beta 1-Cg-Rp\right)\\ \;+\left(1-x\right)\left(1-z\right)\left(Fi+Rg+Ws-Cg\right)\\ Y22=xz\left(Fi+\beta 2-Rp\right)+x\left(1-z\right)Fi\\ \;+\left(1-x\right)z\left(Fi+\beta 2-Rp-Fg\right)+\left(1-x\right)\left(1-z\right)Fi\\ \bar{Y2}=yY21+\left(1-y\right)Y22 \end{array}$$

The replication dynamics equation for the government sector is:


$$\begin{array}{l} Y\left(y\right)=dy/dt=y\left(Y21-\bar{Y2}\right)=y\left(y-1\right)\\ \;\left[Cg-Rg-z\left(\beta 1-\beta 2\right)-\left(1-x\right)zFg-\left(1-x\right)Ws\right] \end{array}$$

The first order derivatives of *y* and the set *J*(*z*) are, respectively:


$$\begin{array}{l} d\left(Y\left(y\right)\right)/dy=\left(2y-1\right)[Cg-Rg-z\left(\beta 1-\beta 2\right)\\ \;-\left(1-x\right)zFg-\left(1-x\right)Ws]\\ J\left(z\right)=Cg-Rg-z\left(\beta 1-\beta 2\right)-\left(1-x\right)zFg-\left(1-x\right)Ws \end{array}$$

**Figure 2:**
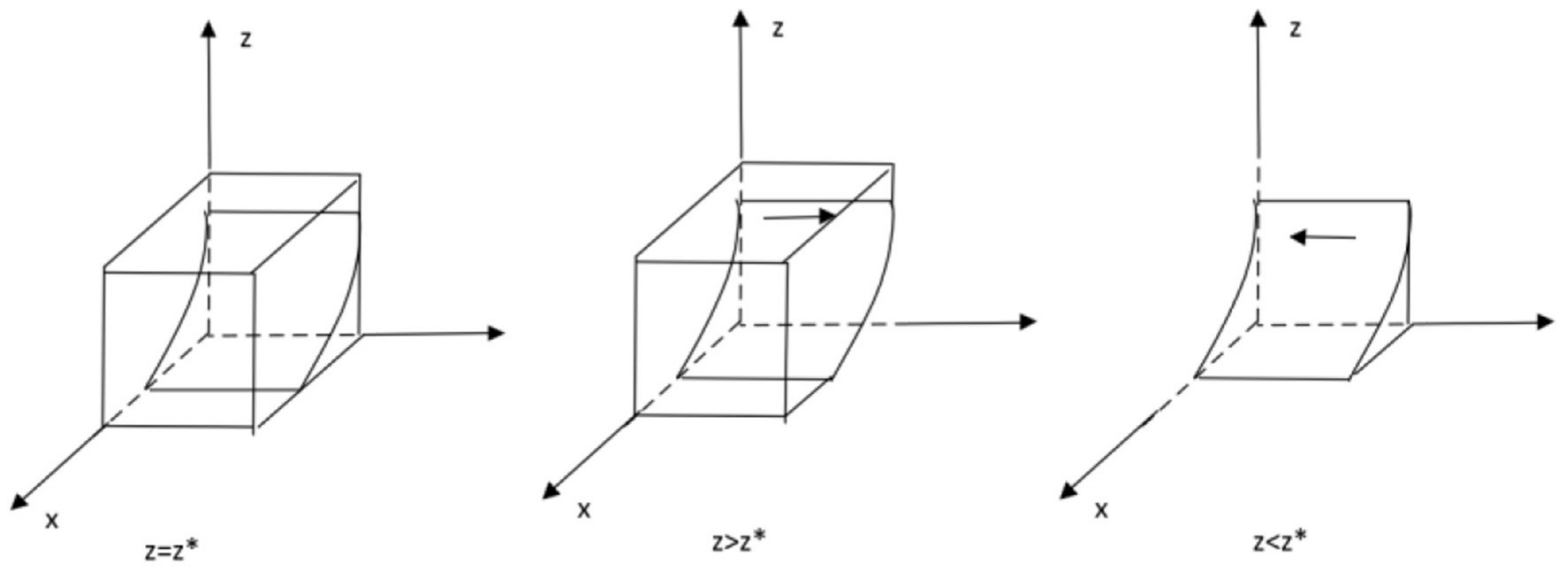
Phase diagram of the evolution of government sector strategies.

From the stability theorem of the differential equation, it follows that the probability of a government department choosing to regulate must be in a steady state: *Y*(*y*) = 0 and *d*(*Y*(*y*))/*dy* < 0. Because ∂*J*(*z*)/∂*z* < 0 therefore *J*(*z*) is a decreasing function with respect to *z*. Therefore when *z* = [*Rg* − *Cg* + (1 − *x*)*Ws*]/[(*x* − 1)*Fg* − (β1 − β2)] = *z*∗,*J*(*z*) = 0. at this point *d*(*F*(*y*))/*dy* ≡ 0, the government department cannot determine a stabilization strategy. When *z* < *z*∗, *J*(*z*) > 0, at this point *d*(*Y*(*y*))/*dy*|*y* = 0 < 0, at this point, *y* = 0 is the evolutionary stabilization strategy for the government sector. Conversely when *z* > *z*∗, *J*(*z*) < 0, *d*(*Y*(*y*))/*dy*|*y* = 1 < 0, at this point, *y* = 1 is the evolutionary stability strategy of the government sector. The evolutionary phase diagram of the government sector's strategy is shown in the following Figure 2.

### 3.4. Public strategy stability analysis

The expected benefits of public scrutiny, no scrutiny and the average expected benefits are:


$$\begin{array}{l} Y31=xy\left(Rp-Cp\right)+x\left(1-y\right)\left(Rp-Cp\right)\\ \;+\left(1-x\right)y\left(Rp-Cp-R1+P\right)\\ \;+\left(1-x\right)\left(1-y\right)\left(Rp-Cp-R1+P\right)\\ Y32=\left(1-x\right)y\left(-R1\right)+\left(1-x\right)\left(1-y\right)\left(-R1\right)\\ \bar{Y3}=zY31+\left(1-z\right)Y32 \end{array}$$

The replication dynamics equation for the public is:


$$\begin{array}{l} Y\left(z\right)=dz/dt=z\left(Y31-\bar{Y3}\right)=z\left(z-1\right)\left[Cp-Rp-P+Px\right] \end{array}$$

The first order derivative of *z* and the set *D*(*x*) are:


$$\begin{array}{l} d\left(Y\left(\text{z}\right)\right)/dz=\left(2z-1\right)\left[Cp-Rp-P+Px\right]\\ \;D\left(x\right)=Cp-Rp-P+Px \end{array}$$

**Figure 3:**
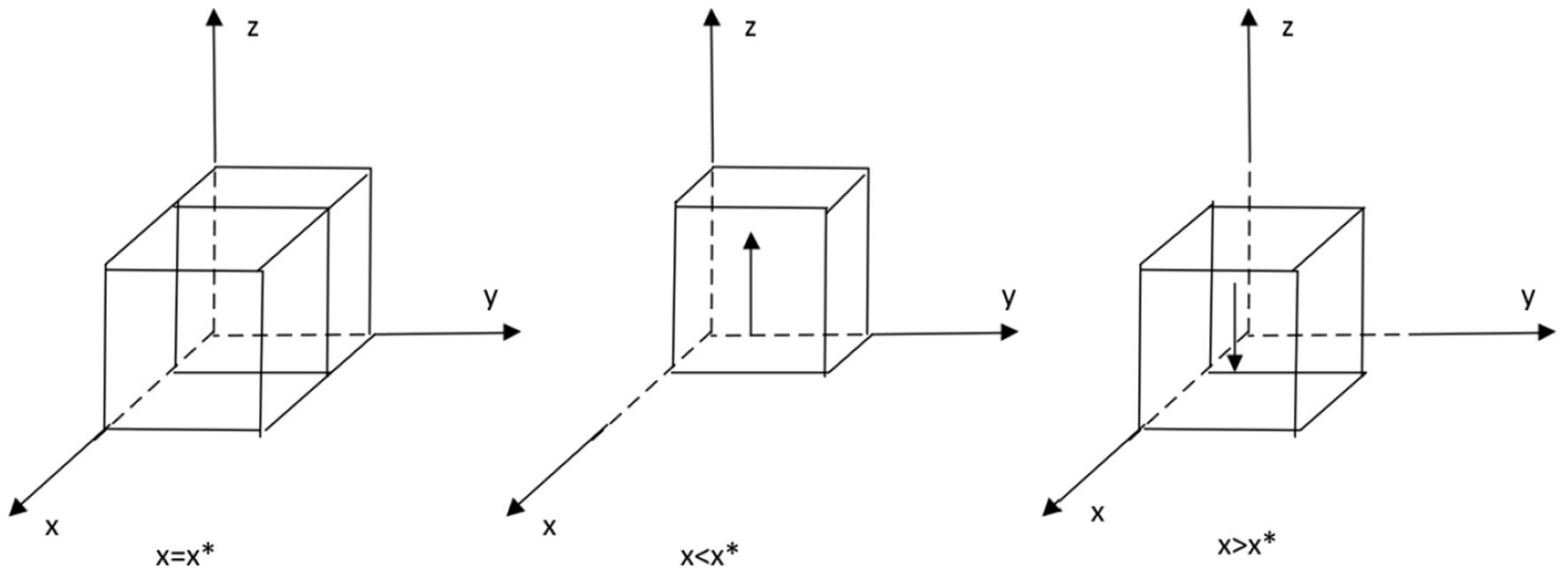
Phase diagram of the evolution of the public's strategy.

It follows from the stability theorem of the differential equation that the probability of the public choosing to monitor must be in a steady state if it is to satisfy:*Y*(*z*) = 0 and*d*(*Y*(z))/*dz* < 0. Because ∂*D*(*x*)/∂*x* > 0 therefore *D*(*x*) is an increasing function with respect to *x*. Therefore, when *x* = (*P* + *Rp* − *Cp*)/*P* = *x*∗, *D*(*x*) = 0, at this point *d*(*Y*(z))/*dz* ≡ 0, the public cannot determine a stabilization strategy. When *x* > *x*∗, *D*(*x*) > 0, at which point *d*(*Y*(z))/*dz*|*z* = 0 < 0, at which point *z* = 0 is the evolutionary stabilization strategy for the public. Conversely, when *x* < *x*∗, *D*(*x*) < 0, *d*(*Y*(z))/*dz*|*z* = 1 < 0, and at this point, *z* = 1 are the public's evolutionary stable strategies. The evolutionary phase diagram of the public's strategy is shown in the following Figure 3.

### 3.5. Stability analysis of the equilibrium point of a tripartite evolutionary game system

The equilibrium point of the system can be obtained from *Y*(*x*) = 0, *Y*(*y*) = 0, *Y*(*z*) = 0 as *E*1(0, 0, 0), *E*2(1, 0, 0), *E*3(0, 1, 0), *E*4(0, 0, 1), *E*5(1, 1, 0), *E*6(1, 0, 1), *E*7(0, 1, 1), *E*8(1, 1, 1). The Jacobian matrix of the three-party evolutionary game system is:


$$\begin{array}{l} J=\left[\begin{array}{ccc} J1 & J2 & J3\\ J4 & J5 & J6\\ J7 & J8 & J9 \end{array}\right]=\left[\begin{array}{ccc} \partial Y\left(x\right)/\partial x & \partial Y\left(x\right)/\partial y & \partial Y\left(x\right)/\partial z\\ \partial Y\left(y\right)/\partial x & \partial Y\left(y\right)/\partial y & \partial Y\left(y\right)/\partial z\\ \partial Y\left(z\right)/\partial x & \partial Y\left(z\right)/\partial y & \partial Y\left(z\right)/\partial z \end{array}\right] \end{array}$$

$$\begin{array}{l} =\left[\begin{array}{ccc} \left(2x-1\right)\left[CH-CL-We-z\left(\alpha 1-\alpha 2\right)-yWs\right] & -x\left(x-1\right)Ws & -x\left(x-1\right)\left(\alpha 1-\alpha 2\right)\\ y\left(y-1\right)\left(zFg+Ws\right) & \left(2y-1\right)\left[Cg-Rg-z\left(\beta 1-\beta 2\right)-\left(1-x\right)zFg-\left(1-x\right)Ws\right] & y\left(y-1\right)\left[-\left(\beta 1-\beta 2\right)-\left(1-x\right)Fg\right]\\ z\left(z-1\right)P & 0 & \left(2z-1\right)\left[Cp-Rp-P+Px\right] \end{array}\right] \end{array}$$

The analysis of the equilibrium points is shown in Table 3.

**Table 3:** Equilibrium point stability analysis.

**Balancing point**	**Jacobian matrix eigenvalues**		
	λ1, λ2, λ3	**Symbols**	**Stability**
*E*1(0, 0, 0)	*CL* − *CH*+*We, P* − *Cp* + *Rp, Rg* − *Cg* + *Ws*	(×, +, ×)	Unstable
*E*2(1, 0, 0)	*Rg* − *Cg, Rp* − *Cp, CH* − *CL* − *We*	(×, +, ×)	Unstable
*E*3(0, 1, 0)	*Cg* − *Rg* − *Ws, P* − *Cp* + *Rp, CL* − *CH* + *We* + *Ws*	(×, +, +)	Unstable
*E*4(0, 0, 1)	*Cp* − *P* − *Rp*, α1 − α2 − *CH* + *CL* + *We*, β1 − β2 − *Cg* + *Fg* + *Rg* + *Ws*	(−, +, +)	Unstable
*E*5(1, 1, 0)	*Cg* − *Rg, Rp* − *Cp, CH* − *CL* − *We* − *Ws*	(×, +, −)	Unstable
*E*6(1, 0, 1)	*Cp* − *Rp*, β1 − β2 − *Cg* + *Rg*, α2 − α1 + *CH* − *CL* − *We*	(−, +, −)	Unstable
*E*7(0, 1, 1)	*Cp* − *P* − *Rp*, β2 − β1 + *Cg* − *Fg* − *Rg* − *Ws*, α1 − α2 − *CH* + *CL* + *We* + *Ws*	(−, −, +)	Unstable
*E*8(1, 1, 1)	*Cp* − *Rp*, β2 − β1 + *Cg* − *Rg*, α2 − α1 + *CH* − *CL* − *We* − *Ws*	(−, −, −)	*ESS*

*Cp* − *Rp* < 0, β2 − β1 + *Cg* − *Rg* < 0, α2 − α1 + *CH* − *CL* − *We* − *Ws* < 0.

## 4. Simulation analysis

In order to verify the validity of the evolutionary stability analysis, the model was assigned to a realistic situation and simulated using Matlab 2022a software. Regarding the assignment of values, first, according to the equilibrium point analysis in Table 2, it is known that (1,1,1) is a stable point, and in order to satisfy the equilibrium condition, all three eigenvalues of (1,1,1) need to be negative, that is, to satisfy *Cp* − *Rp* < 0, β2 − β1 + *Cg* − *Rg* < 0, α2 − α1 + *CH* − *CL* − *We* − *Ws* < 0. Secondly, reference to the established literature reveals that in order to simplify the operations of the simulation analysis, the values are chosen for ease of handling, provided that the three inequalities mentioned above are satisfied (21–23). The values are assigned as follows: *Cp* = 10, *Rp* = 15, β1 =30, β2 = 5, *Cg* = 30, *Rg* = 20, α1 = 20, α2 = 10, *CH* = 70, *CL* = 30, *We* = 30, *WS* = 80, *Fg* = 100, *P* = 300. With the above assignments, the impact of certain parameters on the process and outcome of the evolutionary game is analyzed.

### 4.1. Private sector sensitivity analysis

**Figure 4:**
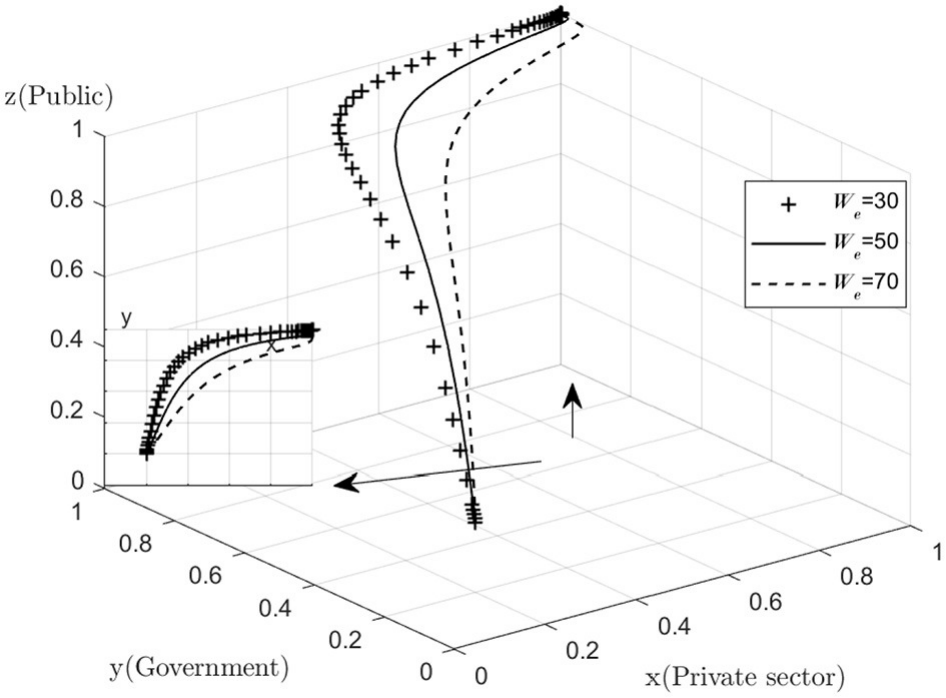
Impact of operating subsidies.

The effect of *We* on the process and outcome of the evolutionary game is analyzed. The simulation results of assigning *We* to *We* = 30, 50, 70*We* = 30, 50, 70, respectively, and replicating the system of dynamic equations evolving 50 times over time are shown in Figure 4. Figure 4 shows that as the system evolves to a point of stability, an increase in government subsidies to private sector operations accelerates the rate of evolution of the private sector to provide high quality services. That is, as *We* increases, the rate at which *x* converges to “1” increases (The *y*–*x* cross section shows that the higher the value of *We*, the closer the value of *x* is to “1” when the same value of *y* is taken). This suggests that government subsidies to the private sector in the combined medical and health-care PPP development process are an important motivation for the private sector's choice to provide high quality services. The above findings are generally consistent with the reality that the higher the subsidy given by the government, the stronger the willingness of the private sector to provide high quality services. The above conclusions are statistically significant, but since the analysis conducted is a simulation, which is more of a measure of the methodology, it lacks a certain degree of comparison with reality. The private sector aims to maximize its own interests, and higher operating subsidies can be used by the private sector to cover the high costs of providing high quality services, but in reality, government funds are limited and the amount of operating subsidies expected by the private sector cannot be fully met. The government should provide appropriate incentives to the private sector when conditions permit, in order to promote the provision of high quality services and sustainable development of combined medical and health-care PPP projects.

**Figure 5:**
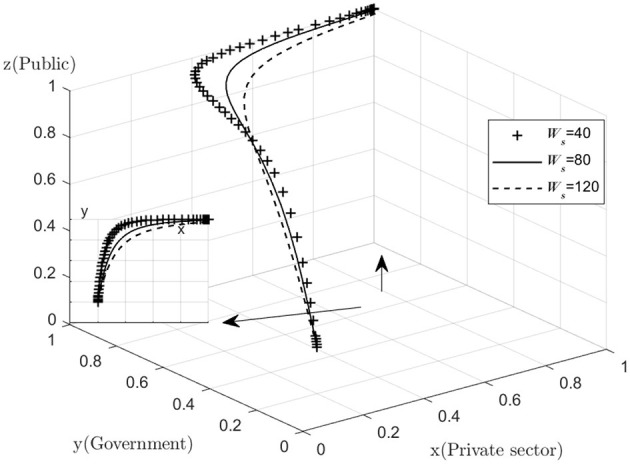
Impact of government fines.

The effect of *Ws* on the process and outcome of the evolutionary game is analyzed. The simulation results of assigning *Ws* to *Ws* = 40, 80, 120*Ws* = 40, 80, 120, respectively, and replicating the system of dynamic equations evolving 50 times over time are shown in Figure 5. Figure 5 shows that as the system evolves to a point of stability, an increase in the default penalty imposed by the government on the private sector accelerates the rate of evolution of the private sector in providing quality services. That is, as *Ws* increases, the rate at which *x* converges to “1” increases (The *y*–*x* cross section shows that the higher the value of *Ws*, the closer the value of *x* is to 1 when the same value of *y* is taken). This suggests that the government should increase the penalties for private sector default in the combined medical and health-care PPP development process, as higher penalties will deter the private sector from stepping over the “red line” of default, and encourage the private sector to provide high quality services. The private sector aims to maximize its own interests, and if the operating subsidy provided by the government does not cover the cost of providing high quality services, the private sector may choose to provide low quality services in order to maximize its own interests.

In the light of the above sensitivity analysis on operating subsidies, the government should offer the private sector a combination of incentives and deterrents by increasing the private sector's operating subsidies where possible, and by setting high penalties for non-compliance so that the private sector is both satisfied with the subsidies and deterred by the severity of the penalties.

### 4.2. Government sensitivity analysis

**Figure 6:**
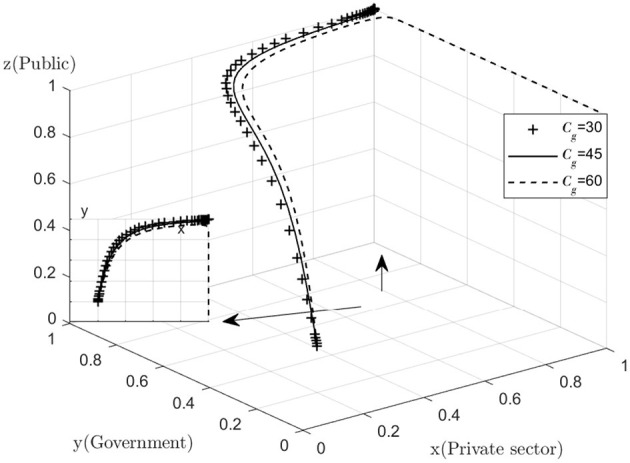
Impact of positive regulatory costs.

Analyze the effect of *Cg* on the process and outcome of the evolutionary game. The simulation results for replicating the system of dynamic equations evolving 50 times over time by assigning *Cg* to *Cg* = 30, 45, 60, respectively, are shown in Figure 6. Figure 6 shows that, as the system evolves to a point of stability, the decrease in the cost of active government regulation speeds up the evolution of government departments from “non-regulation” to “regulation.” That is, as *Cg* decreases, *y* converges to “1” at a faster rate (The *y–x* cross section shows that for the same value of x, the lower the value of *Cg*, the closer the value of *y* is to “1”). This shows that in the process of combined medical and health-care PPP development, as the cost of regulation decreases, the willingness of governments to choose to regulate is significantly enhanced. Since the government's main demand is to pursue social interests and maximize its own interests, if the cost of regulation is too high and the additional benefits of regulation are lower than the cost of regulation, the government may reduce its willingness to actively regulate. If the combined medical and health-care PPP project is left unregulated for a long period of time, there is no guarantee that the private sector will choose to provide high quality services in order to maximize their own interests, resulting in a low quality of operation of the combined medical and health-care PPP project. If the public finds that the government is not actively regulating, they may give the government a lower level of reputation and the government's reputation will be damaged. In the long run, the combined medical and health-care PPP project may end up as a failure with minimal overall social utility. However, for a number of reasons, the government is unable to significantly reduce the cost of regulation in the short term while ensuring the quality of regulation, so the government needs to consider the overall interests of society and its own reputation and insist on active regulation.

**Figure 7:**
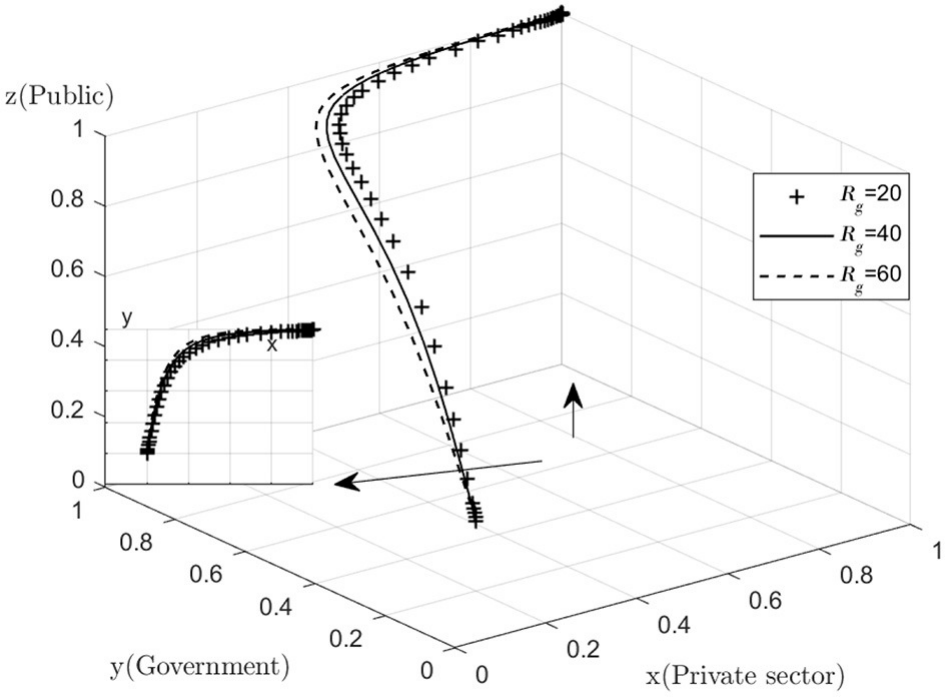
Impact of additional regulatory benefits.

Analyze the effect of *Rg* on the process and outcome of the evolutionary game. The simulation results for replicating the system of dynamic equations evolving 50 times over time by assigning *Rg* to *Rg* = 20, 40, 60, respectively, are shown in Figure 7. Figure 7 shows that, as the system evolves to a point of stability, the increase in the additional benefits of active government regulation speeds up the evolution of government departments from “no regulation” to “regulation.” That is, as *Rg* increases, the rate at which *y* converges to “1” increases (The *y–x* cross section shows that for the same value of x, the higher the value of *Rg*, the higher the value of *y* tends to “1”). This suggests that the willingness of the government to choose to engage in active regulation has increased significantly as the additional benefits gained from active government regulation increase. If the additional benefits of regulation are too small to compensate for the high cost of active regulation, the government may be less willing to actively regulate. If the public finds that the government is not actively regulating, they may give the government a lower level of reputation, which may result in the government's reputation being damaged. However, for many reasons, the regulatory subsidies received by the government will not increase significantly in the short term, so the government needs to consider more about the overall interests of society and its own credibility level and insist on active regulation.

### 4.3. Public sensitivity analysis

**Figure 8:**
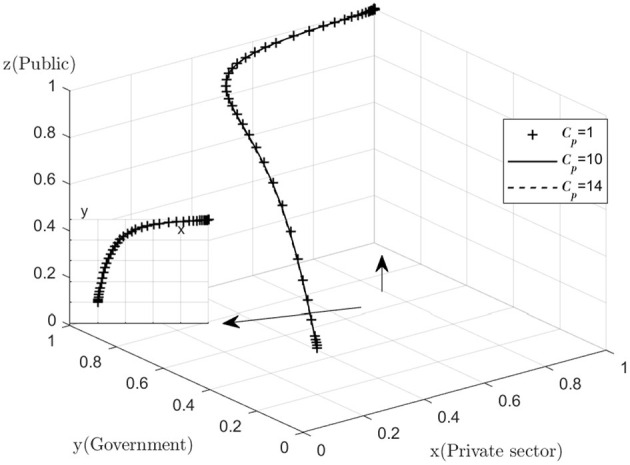
Impact of active monitoring costs.

**Figure 9:**
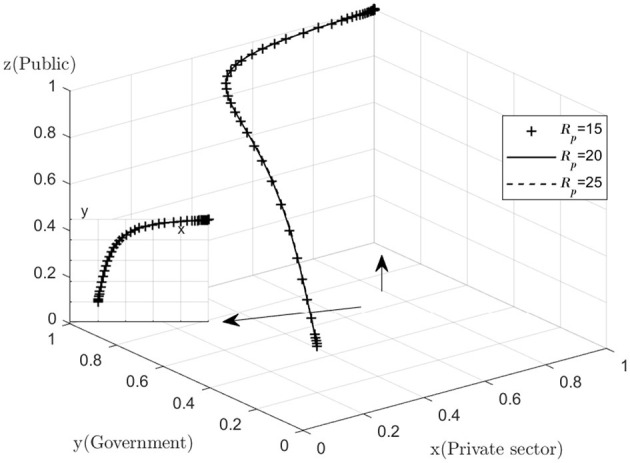
Impact of active supervision rewards.

With regard to the sensitivity analysis of the public, as the changes in quantities *Cp, Rp* are too subtle. Therefore, the graph does not show a significant difference, but a common sense analysis shows that the decrease in the cost of active monitoring and the increase in the reward for active monitoring will help the public to increase their willingness to actively monitor. The public's goal is to maximize their own interests, and those who will monitor the combined medical and health-care PPP project are mostly consumers of the combined medical and health-care PPP project and their relatives, and it is the quality of the combined medical and health-care PPP project that they care most about. No matter how the value of *Cp, Rp* is varied, the difference in the final operating results is always small (Figures 8, 9).

## 5. Discussion

The most desirable combination of strategies for the three parties evolutionary game is (1, 1, 1), where the private sector provides high quality services, the government actively regulates and the public actively participates in monitoring. The following is an analysis of the respective evolutionary outcomes of the three parties.

The results of the private sector simulations show that the rate at which the private sector evolves toward “1” is determined by a combination of government subsidies *We* for high quality services and government fines *Ws* for low quality services. The private sector, aiming to maximize its own interests, if the government subsidies its operations sufficiently, or penalizes it sufficiently, or both, the private sector is likely to choose to provide high quality services as an incentive and as a deterrent. In the case of the private sector, the government should be able to give the private sector both incentives and deterrents, while at the same time recognizing the deterrent effect of the penalties.

In combined medical and health-care PPP projects, the private sector often adopts two approaches to improve existing operations in order to increase the project's operating returns: the first is to improve the quality of health care services through internal optimization so that operating costs can be reduced, and the second is to reduce the quality of health care services to achieve a reduction in costs and thereby make a profit (24). The first approach requires significant resources to improve internal management practices and pioneer technological innovation while maintaining the current service prices, while the second approach is opportunistic and may lead to a loss of interest for the public. The more subtle the public's perception of changes in the quality of the services provided by combined medical and health-care PPP project, the less scope there is for the private sector to make profits by cutting quality while keeping the price of the services the same.

The simulation results for the government show that the cost of active government regulation *Cg* and the additional subsidies received by the government for active regulation *Rg* together determine the rate at which the government's evolutionary strategy evolves toward “1.” If the cost of regulation is too high or if the subsidies received from active regulation are far from covering the cost of active regulation, the government's willingness to actively regulate will decline.

Under the combined medical and health-care PPP model, although government departments can improve the incompleteness of information by taking a small percentage of shares in the SPV (Special-Purpose-Vehicle), their role is limited after all. The threshold of expertise in the combined medical and health-care sector is high, and government departments need to continuously invest more resources and actively supervise in order to fully access the true operation of combined medical and health-care PPP projects.

Most combined medical and health-care PPP have an operational cycle of 15–20 years, while most local administrators are in office for only 5 years. This time mismatch may result in a situation where one administrator leaves the risks to the next, triggering intergenerational injustice. In the traditional GDP(Gross Domestic Product)-led promotion mechanism, basic projects can effectively incentivize local administrators with easily measurable results (25), yet by the time the project enters the operational phase, the local government department to which the combined medical and health-care PPP project belongs will have long since changed. As a result, the distorted incentive of administrative promotion has more or less inhibited the motivation of government departments to actively regulate.

Although it is not possible to determine changes in the state of evolution directly from the results of the public's simulation, it is important to recognize both the costs *Cp* and the rewards*Rp* of active public monitor. If the cost of monitoring is too high, this may also reduce the willingness of the public to monitor to a certain extent.

Combined medical and health-care PPP projects are inherently complex, and in most cases faced with the dilemma of involving multiple actors in management. By participating in monitoring and providing feedback, the public can identify gaps in the management and quality of services provided by the government and the private sector. The public is in a weaker position in the game than the government and the private sector, and although they can report or defend their rights against poor quality services through online disclosure platforms, but often with a longer waiting period. This has led to two types of negative behavior: firstly, some members of the public prefer to accept low quality health-care services rather than spend time and money to defend their rights, and secondly, the occurrence of extreme behavior such as “medical malpractice,” which has a negative impact on the good functioning of public order and may also affect the health and well-being of individuals, may be followed by the rest of the public if not dealt with properly. Therefore, there is a high probability that the public will choose to actively monitor the situation, but the cost of defending their rights after the monitoring becomes a key factor in whether the public will carry out the monitoring to the end. At this point, appropriate motivations from the government may stimulate the public's willingness to defend their rights, prompting them to carry out their rights to the end, and then promote the development of combined medical and health-care PPP projects in the direction of quality.

## 6. Case studies

The Hongjiang People's Hospital Tongxin Medical and Health-Care Centre PPP Project is located in Hongjiang, Huaihua City, Hunan Province. The project is operated under the ROT (Rehabilitate-Operate-Transfer) model, in which the Hongjiang People's Hospital is authorized by the Hongjiang Municipal Government and appointed to co-finance the project company with the private sector, also known as SPV. The Hongjiang Municipal Government and the SPV signed the PPP Project Contract, granting the SPV the concession to undertake the construction, operation, maintenance and renewal and replacement of the project during the cooperation period.

**Figure 10:**
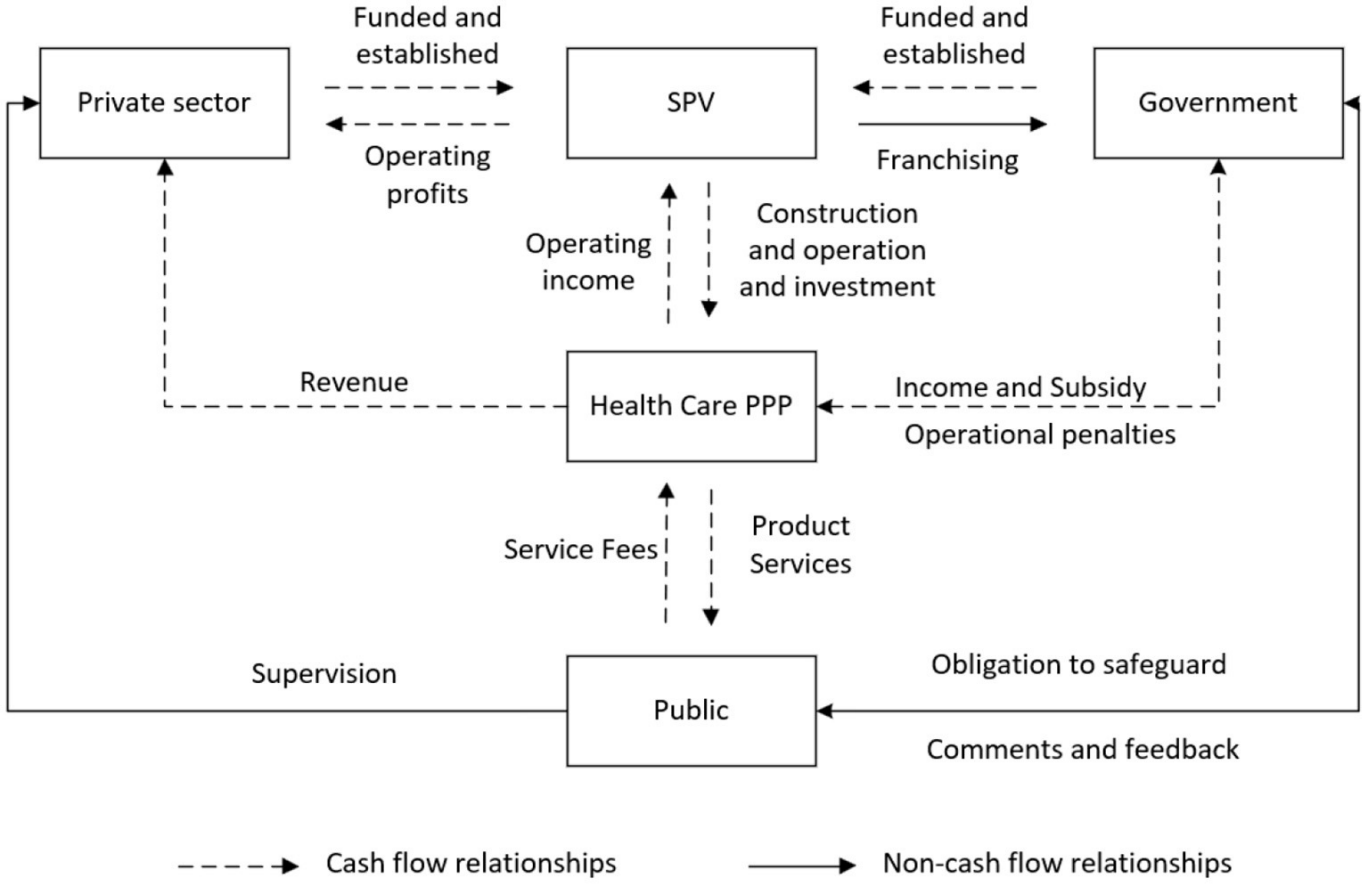
Map of the interests of the three parties.

The project is a profit-making medical and health-care project. During the cooperation period, the project company is responsible for operating the project for 30 years to recover the investment and obtain reasonable income. At the same time, the government will regulates and assess the operation and maintenance of the project during the partnership period, and discipline the project company to ensure the quality of service through performance assessment. The public also monitors the actions of the government, the private sector and the SPV company to ensure that their interests are maximized. The following diagram shows the relationship between the interests of the three parties involved in the PPP project for the Tongxin Medical and Health-Care Centre of the Hongjiang People's Hospital (Figure 10).

## 7. Conclusions and recommendations

This paper investigates the behavioral strategies of the three parties in the process of PPP regulation of health-care integration, and analyzes the evolutionary stabilization strategies of the three parties by constructing an evolutionary game model. The above simulation results show that there are two ways to increase the willingness of the private sector to provide high quality services, either by increasing the amount of government subsidies to the private sector or by increasing the level of government penalties to the private sector. Moreover, the motivation for government departments to regulate depends heavily on the cost of regulation or the benefits of regulation. At the same time, the ability of the public to regulate combined medical and health-care PPP projects depends largely on incentives. The three parties' ultimate ideal situation is that the private sector provides high quality services, the government actively regulates, and the public actively participates in monitoring. This paper enriches the study of game behavior strategies among multiple subjects in the field of health care combination, and lays the foundation for subsequent game studies.

At present, the government has put forward relevant regulations on possible problems in regulation, such as requiring that both construction and operation costs of projects borne by the government should be paid based on the results of performance assessment, and that the portion of construction costs involved in performance assessment should not be < 30%. In order to further improve the quality of regulation of combined medical and health-care PPP projects and promote the sustainable development of combined medical and health-care PPP projects, this paper puts forward the following recommendations:

1. Establish an independent regulator, broaden public feedback channels and improve PPP-related laws (26). Establish a regulatory body that is independent of the government and the private sector and runs through the entire life cycle of combined medical and health-care PPP projects (27). The regulator will have independent regulatory and approval functions, and can impose uniform administrative penalties on the private sector and local governments for dereliction of duty. The existing public feedback channels can be broadened, and a PPP credit platform can be established for the whole society, in the form of an app or public number as a carrier, and the private sector can be included in the credit “blacklist” for dereliction of duty, thus increasing the cost of their opportunism.
2. Innovate project operation methods and enhance the operational capacity of enterprises. At present, most of the combined medical and health-care PPP models in the market are newly built, and there is a large backlog of stock projects (28). The institutional inertia of the BOT (Build-Operate-Transfer) model, which has been used more often in the past for combined medical and health-care PPP projects, has led to the private sector's main source of profit being the construction phase, lacking the incentive to innovate in form. Therefore, government departments can encourage the private sector to carry out combined medical and health-care PPP projects in the form of TOT (Transfer-Operate-Transfer), LOT (Lease-Operate-Transfer) and ROT (Rehabilitate-Operate-Transfer), and further revitalize existing health-care resources by integrating and transforming them (29).

Although this paper has studied the evolutionary game of the three parties players in the PPP of combined medical and health-care, it is less relevant to the actual situation because it is a simulation analysis, and further integration with actual cases and introduction of algorithmic models into the field of health-care combination can be considered in the future (30, 31). Meanwhile, this paper only studies the interest relationship between the government, the private sector and the public. In the future, intermediate organizations can be introduced to form a quadrilateral interest relationship and conduct a quadrilateral evolutionary game study.

## Data availability statement

The original contributions presented in the study are included in the article/supplementary material, further inquiries can be directed to the corresponding author.

## Author contributions

MZ: conceptualization, methodology, formal analysis, and writing—original draft. PY: study supervision. Both authors contributed to the article and approved the submitted version.
